# Geometric Phase Generated Optical Illusion

**DOI:** 10.1038/s41598-017-11945-z

**Published:** 2017-09-12

**Authors:** Fuyong Yue, Xiaofei Zang, Dandan Wen, Zile Li, Chunmei Zhang, Huigang Liu, Brian D. Gerardot, Wei Wang, Guoxing Zheng, Xianzhong Chen

**Affiliations:** 1 SUPA, Institute of Photonics and Quantum Sciences, School of Engineering and Physical Science, Heriot-Watt University, Edinburgh, EH14 4AS UK; 2 Shanghai Key Lab of Modern Optical System, University of Shanghai for Science and Technology, Shanghai, 200093 China; 3 School of Electronic Information, Wuhan University, Wuhan, 430072 China; 4 Tianjin Key Laboratory of Optoelectronic Sensor and Sensing Network Technology, College of Electronic Information and Optical Engineering, Nankai University, Tianjin, 300350 China

## Abstract

An optical illusion, such as “Rubin’s vase”, is caused by the information gathered by the eye, which is processed in the brain to give a perception that does not tally with a physical measurement of the stimulus source. Metasurfaces are metamaterials of reduced dimensionality which have opened up new avenues for flat optics. The recent advancement in spin-controlled metasurface holograms has attracted considerate attention, providing a new method to realize optical illusions. We propose and experimentally demonstrate a metasurface device to generate an optical illusion. The metasurface device is designed to display two asymmetrically distributed off-axis images of “Rubin faces” with high fidelity, high efficiency and broadband operation that are interchangeable by controlling the helicity of the incident light. Upon the illumination of a linearly polarized light beam, the optical illusion of a ‘vase’ is perceived. Our result provides an intuitive demonstration of the figure-ground distinction that our brains make during the visual perception. The alliance between geometric metasurface and the optical illusion opens a pathway for new applications related to encryption, optical patterning, and information processing.

## Introduction

Optical illusions, such as “Fraser spiral illusion”, “Nuts illusion”, and “Rubin’s vase”, are characterized by visually perceived images that are deceptive or misleading, violating the saying “seeing is believing”. Traditional optical illusions are typically realized by using specific visual tricks, i.e., complicated graphic design, or under extreme natural environment such as mirages, meaning that they are mainly demonstrated in macroscopic scale. The realization of optical illusions based on optical nanodevices with high resolution has not been demonstrated. Metasurfaces^1–3^, a new subtype of metamaterials, consisting of a thin layer of plasmonic or dielectric nanostructures have attracted considerable attention in nanophotonics due to their unique capability of manipulating electromagnetic wavefront at subwavelength resolution in a desirable manner. Various types of metasurfaces have been proposed and designed to realize novel optical functionalities such as generalized Snell’s law of refraction^1, 2, 4, 5^, Spin-Hall effect^6^, dual-polarity planar metlens^7^, wave plates^8^, vortex beam generation^9–12^, spin-controlled photonics^13–15^, and unidirectional excitation of surface plasmon polariton^16^.

Computer-generated holograms (CGH)^17–19^ offer important advantages over optical holograms since there is no need for a real object. A holographic image can be generated by digitally computing a holographic interference pattern and encoding it into a specific surface structure or a spatial light modulator for subsequent illumination by suitable coherent light source. Benefiting from the unprecedented manipulation of light propagation due to the desired phase change at the interface, metasurfaces have been employed for the application of holography^20–23^, including highly efficient broadband holograms^11, 24, 25^, image-switchable holograms, full-color holograms^26–28^ and nonlinear holograms^29^. With the advancement of nanotechnology and integrated photonics, miniaturization and integration are the two main tireless pursuits for the production of optical devices. To date, all of the demonstrated metasurface holograms are based on the phase profile to generate the corresponding holographic image. How to generate an additional visual image based on the same ultrathin metasurface device without its closely related phase profile, which can be considered as an optical illusion, has not been demonstrated.

In this paper, we propose and experimentally demonstrate an approach to realize an optical illusion based on a metasurface. The most famous example of figure-ground perception is probably the vase-face drawing that Edgar Rubin described. The brain usually identifies an object by distinguishing the shape or figure from the background. The perceived image in brain depends critically on which border is assigned. If we create two separated faces regions with central symmetric distribution, a shape of vase is perceived (optical illusion) because the human visual system settle the faces as background. We take this drawing as an example for demonstration. The metasurface device is designed to display two asymmetrically distributed off-axis images of “Rubin faces” with high fidelity and a wide field of view. Upon the illumination of a linearly polarized light beam, the optical illusion of “vase” can be perceived. The reflective-type metasurface consisting of metallic nanorod array and ground metallic film with the dielectric layer sandwiched between them, is used to generate Pancharatnam-Berry (P-B) phase over a broad range of wavelengths with high efficiency. The realization of optical illusion with metasurface represents a unique application where metasurfaces can better show their superior performance due to the generated geometric phase at the interface. The optical illusion that we demonstrated is caused by the information gathered by the eye, which is processed in the brain to give a perception that does not tally with a physical measurement of the stimulus source. This type of stimulus is of great interest and importance since it provides a marvelous and intuitive demonstration of the figure-ground distinction the brain makes during visual perception.

## Results

**Figure 1:**
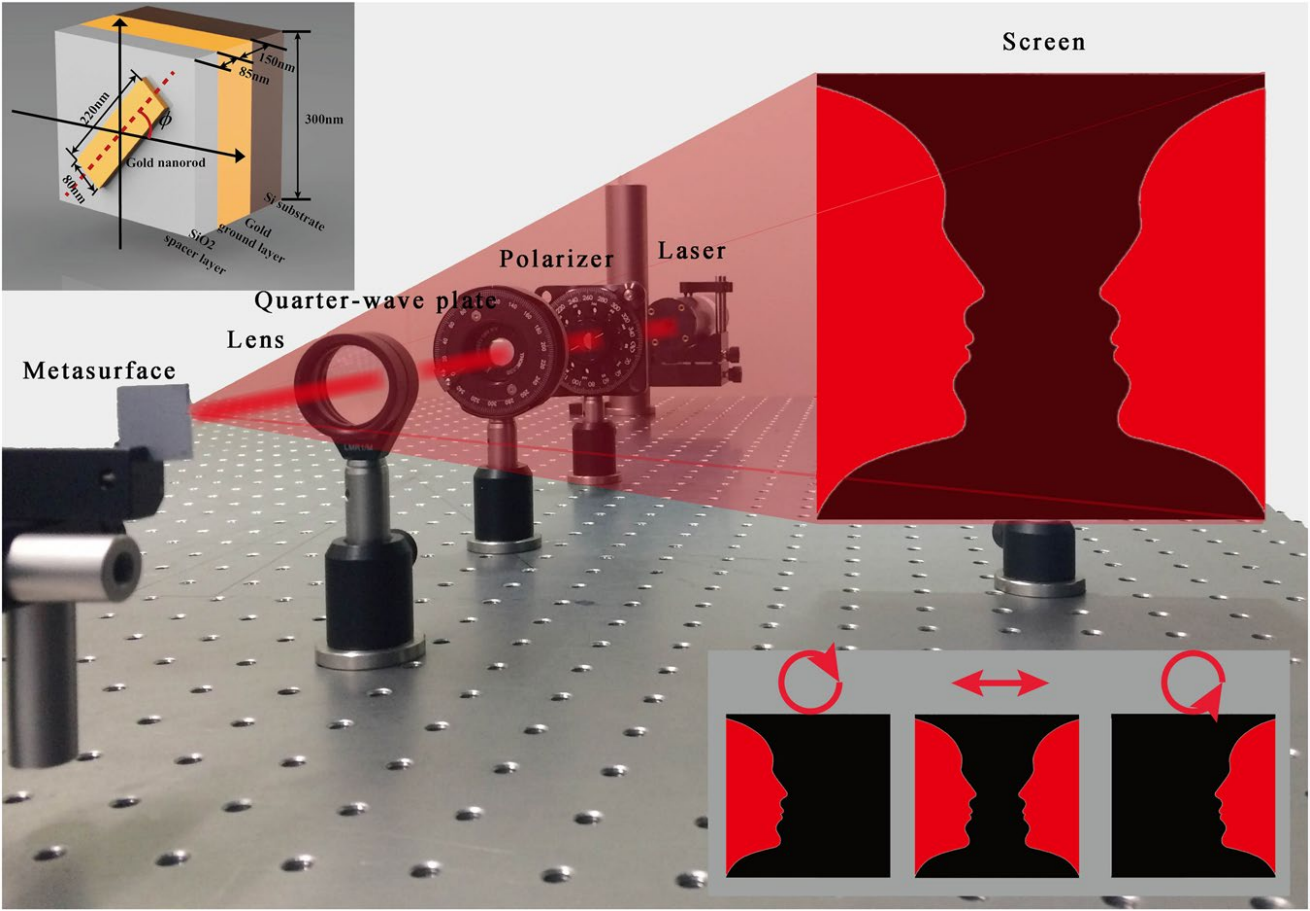
Schematic diagram of the experimental setup to generate optical illusions. A polarizer and a quarter-wave plate are used to generate the incident light with required polarization states. Upon the illumination of linearly polarized light at normal incidence, an optical illusion is perceived on the viewing screen. The top-left inset shows a single pixel of the reflective metasurface, which consists of gold nanorods on the top, a SiO_2_ layer in the middle and a gold layer at the bottom. Under the illumination of RCP and LCP, the ‘Rubin’s face’ (bottom right) is projected onto the left and right side of the image plane, respectively. The ‘vase’ (optical illusion) is perceived between the two separated “Rubin’s faces” during the visual perception.

To improve efficiency and image quality while maintaining the broadband property, we leverage the recent advances in the realization of high efficiency, broadband reflective-type configuration and geometric metasurfaces. In comparison with other types of metasurfaces, a metasurface consisting of nanorods with spatially varying orientation shows superior phase control for circular polarization and can ease the fabrication. Figure 1 shows the schematic of the geometric-phase induced optical illusions. The reflective-type metasurface, consisting of a gold ground layer, a SiO_2_ spacer layer and a top layer of elongated gold nanorods, is utilized to generate the required phase profile (Fig. 1 top left). Each unit cell of the metasurface, containing a subwavelength nanorod with carefully designed azimuthal orientation, can be considered as an anisotropic scatterer. When a circularly polarized light beam is incident onto nanorods, the reflected light consists of two parts: one has the same handedness with an additional phase change (known as P-B phase), and the other has the opposite handedness without phase change^22^. By carefully controlling the orientation of the nanorods, the desired continuous phase profile with constant amplitude can be achieved. As shown in Fig. 1 (bottom right), an off-axis “Rubin face” located at left side or right side of the viewing screen can be reconstructed upon the illumination of right-handed or left-handed circularly polarized (RCP or LCP) light. Since a linearly polarized light beam can be decomposed into two opposite circularly polarized light beam with equal components, an additional image named “vase” without encoding the corresponding phase profile onto the designed metasurface can be perceived between the two faces. When two reconstructed images have a common border, and one is seen as figure (“Rubin face”) and the other as ground (“vase”), the immediate perceptual experience is characterized by a shaping effect which emerges from the common border of the fields and which operates only on one image or operates more strongly on one than on the other.

**Figure 2:**
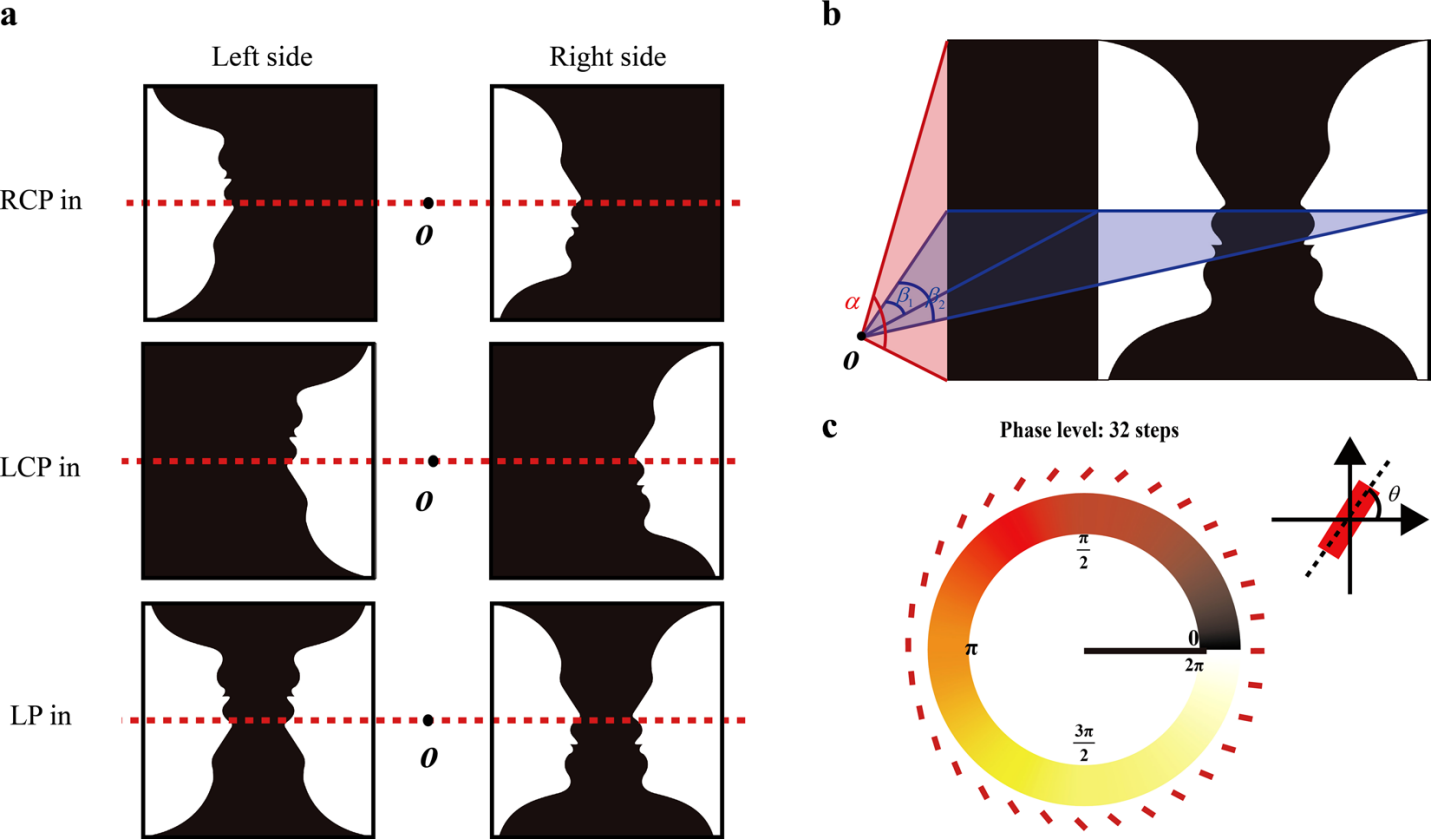
Mechanism for the realization of optical illusions and the schematic of the design. (**a**) Generation of the optical illusion. Two asymmetrically distributed ‘Rubin faces’ (one upright and one inverted) are designed for the incident light with circular polarizations. Upon the illumination of RCP light, the upright and inverted ‘Rubin faces’ are generated on the right and left sides of the viewing plane, respectively. The two ‘Rubin faces’ will be rotated 180° and swapped due to the phase-conjugation and the spin-orbit coupling if the helicity of the incident light is changed from RCP to LCP. Two pairs of ‘Rubin faces’ (one upright, one inverted) are reconstructed upon the illumination of incident light with linear polarization since a linearly polarized light beam can be decomposed into LCP and RCP light beams with same components. When two reconstructed images have a common border, and one is seen as figure (“Rubin faces”) and the other as ground (“vase”), the immediate perceptual experience is characterized by a shaping effect emerging from the common border of the field. (**b**) Geometric parameters of the projected images that correspond to the designed hologram. The off-axis angle *β*_1_ is 9.75°. The target image angles *α* and *β*_2_ are designed to be 23° and 30°, respectively. (**c**) Phase delay for the different phase levels. 32 phase levels (−*π* to *π* with the interval of *π*/16) are used in the design. Each elongated nanorod is rotated along *x* axis to achieve the desired local phase.

**Figure 3:**
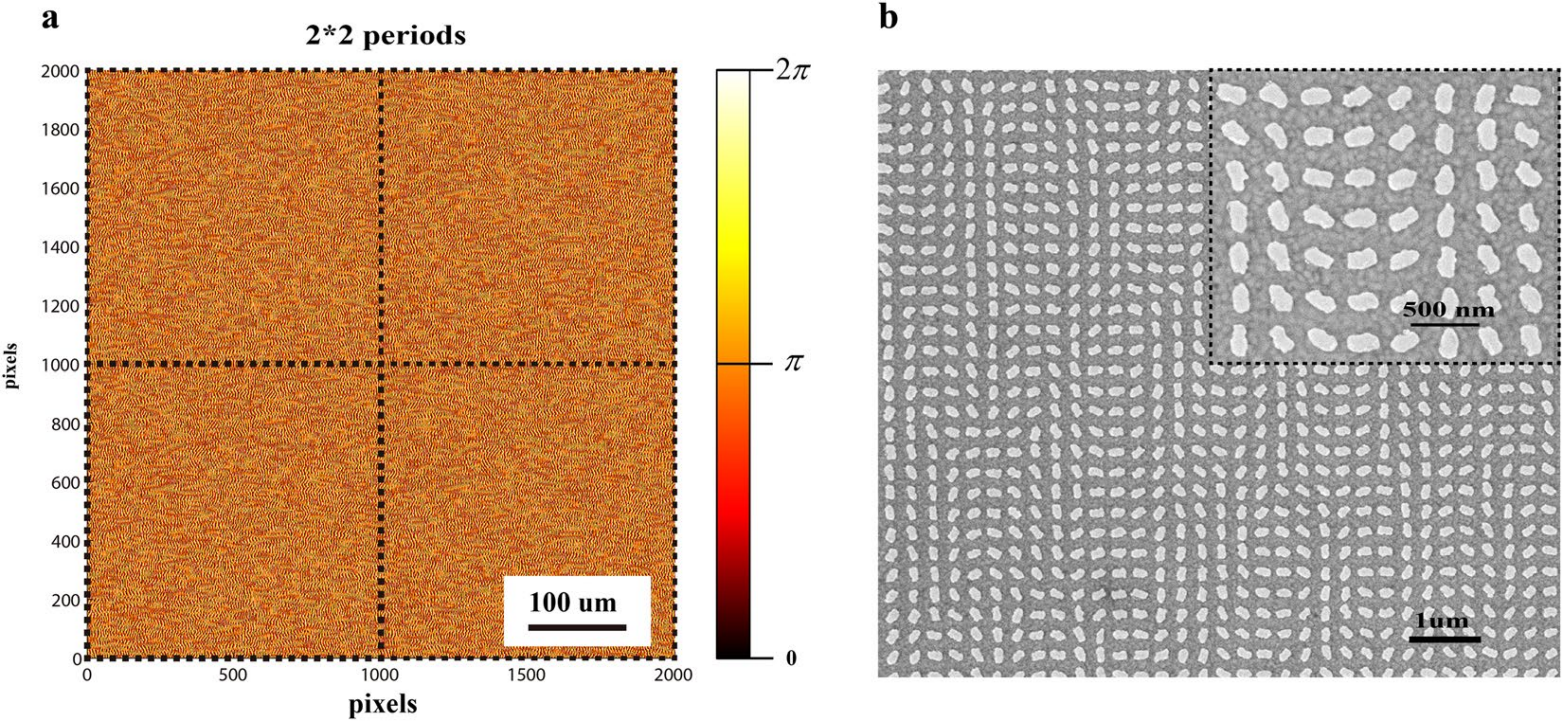
The phase distribution and the fabricated sample. (**a**) The calculated 32-level phase distribution with 2 × 2 periods. (**b**) Scanning electron microscopy (SEM) image of part of the fabricated metasurface device.

Unlike the previous polarization multiplexed metasurface holograms with symmetrically distributed target images^22, 23^, the two off-axis “Rubin faces” are designed asymmetrically, as shown in Fig. 2a. For RCP light illumination, two “Rubin faces” (one upright and one inverted) are reconstructed on the two sides of the zero-order spot. While for the LCP light illumination, the two “Rubin faces” are rotated 180° counterclockwise and horizontally flipped around point *O* due to the phase-conjugation induced by different helicity of the incident light. For the case of linearly polarized light which can be decomposed into LCP and RCP light with same components, the upright and inverted “Rubin’s vase” illusions are generated on the both sides. The Gerchberg-Saxton algorithm is utilized to obtain the expected phase profile of the phase-only hologram. The target image is discretized as a number of pixels in which each pixel is regarded as a point source. The design method can be found in Methods. By encoding the phase of the hologram into the metasurfaces, the target images are reconstructed under the illumination of properly polarized light. Our hologram is designed with an off-axis angle of *β*_1_ = 9.75° and a large field of view of 30° × 23° along horizontal and vertical directions, respectively (Fig. 2b). Although arbitrary phase levels can be achieved since the encoding process from the phase profile into pixelated nanoantennas is very straightforward, we choose 32-phase levels (Fig. 2c) instead of continuous phase distribution to minimize the near field coupling between neighbouring nanorods. Here, a 2 × 2 periodic array of the phase (“Rubin face”) pattern with pixel size of 300 nm × 300 nm and pixel number of 2000 × 2000 is designed to improve the fidelity of constructed image (see Fig. 3a)^22^. Based on the concept of Dammann grating, the 2 × 2 periodic array design can improve the image quality by reducing the effect of laser speckle in the reconstructed images (see Supplementary Section 1). The whole size of sample is 600 um. A 150-nm-thick gold film and a 85-nm-thick glass spacer are deposited on the silicon substrate by electron beam evaporation. The length, width and thickness of each nanorod are 220 nm, 80 nm and 30 nm, respectively. The nanorod structure is fabricated using standard electron beam lithography and a subsequent lift-off procedure. The detailed fabrication process is given in Supplementary Section 2. The scanning electron microscopy (SEM) image of the fabricated metasurface consisting of nanorods with spatially varying orientation is shown in Fig. 3b.

**Figure 4:**
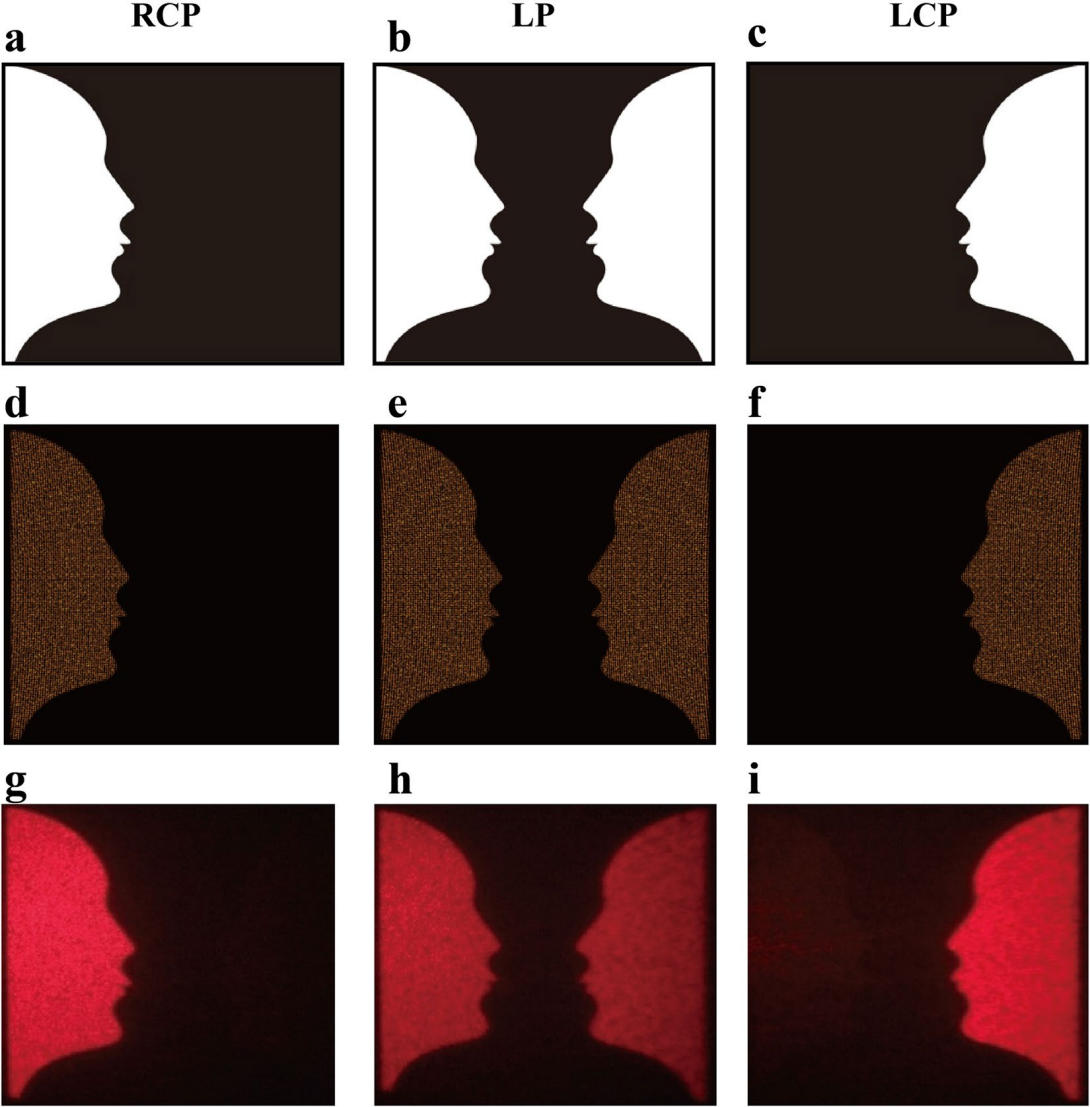
Reconstructed images versus incident polarization states at 633 nm. The figures in the first and second rows represent target images and simulated results, respectively. While the figures in the third row represent the corresponding experimental results. The polarization states of the incident light are chosen to be RCP for (**a**), (**d**) and (**g**), linear polarization for (**b**), (**e**) and (**h**), and LCP for (**c**), (**f**) and (**i**), respectively.

Figure 4 shows the target images, simulation results and corresponding experimental results upon the illumination of incident light with different polarization states. Figure 4a–c illustrate the original target images (“Rubin faces”), depending on the polarization states of the incident light. These target images of “Rubin face” or “optical illusion (vase)” can be simulated by considering light emission from all the discretized point sources, as shown in Fig. 4d–f. Experimentally, a polarizer and a quarter-wave plate are located behind the tunable laser source (NKT, SuperK EXTREME) to generate the required polarized states. Then, the light beam with a beam size of 2 mm is focused by using a plano-convex lens (*f* = 150 mm) and incident onto the fabricated sample (Fig. 3b). Two off-axial holographic images are reconstructed at the normal incidence. Here, a viewing screen is used to display holographic images. Figure 4g–i show the experimentally captured holographic images for different polarization states of the incident light at the wavelength of 633 nm. The distance between the screen and the metasurface is 60 mm. Upon the illumination of RCP light, a holographic image named “Rubin face” with high signal-to-noise is reconstructed on the left side of the screen (Fig. 4g). It should be noted that the size of the “Rubin face” is proportional to the reconstructed distance between the sample and the screen. When the polarization of incident beam is changed from RCP to LCP, a horizontally flipped image of “Rubin face” is displayed on the right side (Fig. 4i), which clearly shows that the position of the holographic image is solely dependent on the helicity of the incident light. LP light can be decomposed into LCP and RCP light with equal components, therefore, two pairs of different centrosymmetric “Rubin faces” (one upright and one inverted) shown in Fig. 4h are generated. Even more intriguingly, an additional image of “vase” is also perceived between these two “Rubin faces”. It should be mentioned that the “vase” is the optical illusion perceived by our eyes during the visual perception, which has no corresponding phase profile encoded onto the metasurface. The images of the inverted illusions are shown in Supplementary Figure S2.

## Discussion

**Figure 5:**
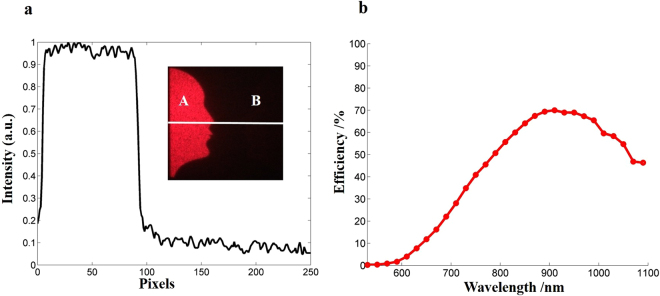
The signal-to-noise ratio (SNR) and conversion efficiency of the fabricated metasurface device. (**a**) The measured SNR of the reconstructed ‘Rubin face’. Area A is a part of the ‘Rubin face’ while area B is the portion of the background. (**b**) The experimentally measured efficiencies over a broad range of wavelengths. The conversion efficiency is defined as the power of two pair of “Rubin faces” divided by that of the incident light.

As a new optical device, its performance is our main concern. Signal-to-noise ratio (SNR) is one of the most critical factors to determine the quality of optical illusion. The SNR here can be defined as the ratio between the mean power of area A and the standard deviation of area B (see Fig. 5a). The calculated SNR is nearly infinity because the power of the background is nearly zero. In experiment, the background noise is mainly caused by the irregularity of nanorods and non-rigid of the plane-wave incidence. The measured SNR of the optical illusion is 7.6 (Fig. 5a), which can be further improved by optimising the fabrication process and optical experimental setup. The conversion efficiency is defined as the ratio of the power of all the reconstructed images and the input power. Here a condenser lens (*f* = 32 mm) is used to collect the generated images. The efficiency was measured over an ultra-broadband super-continuous spectrum in the range from 530 nm to 1090 nm, and it is higher than 45% in a relatively broad spectral ranging from 770 nm to 1090 nm. We achieve the maximum conversion efficiency in experiment is 69.94% at the wavelength of 910 nm. No twin images are observed in our experiment since the pixel size (300 nm) is much smaller than the wavelength of the incident light. The dependence of conversion efficiency and SNR on the wavelength is given in the Supplementary Section 4. In theory, the designed device to reconstructed optical illusion can be worked over a wide range of wavelengths, since the metasurfaces exhibit a dispersion-less phase profile resulting from the geometric P-B phase determined by the orientation of nanorods. The simulated conversion efficiency can be found in Supplementary Section 5. The difference between experimental results and simulation results is mainly due to the titanium layer between nanorods and SiO_2_ layer and the fabrication error of nanorods.

**Figure 6:**
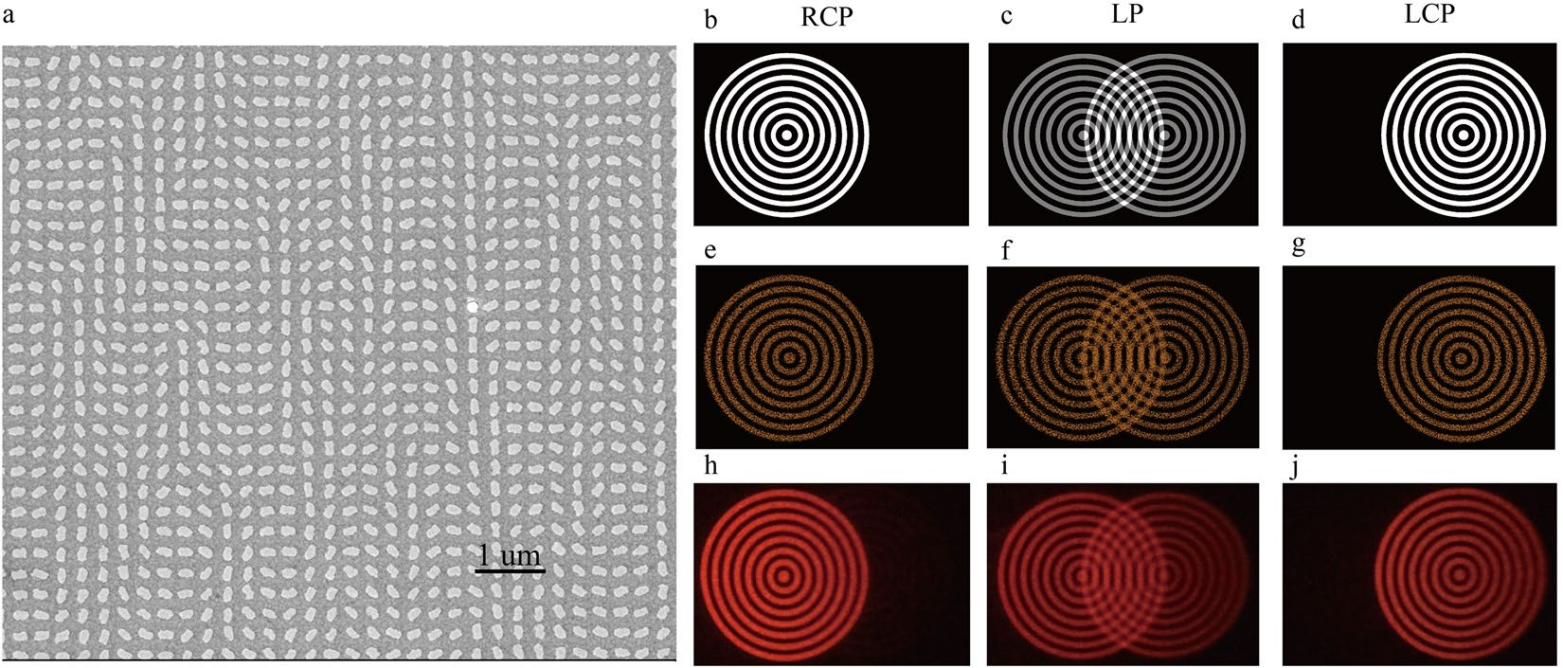
The fabricated sample for Moiré pattern generation and reconstructed images versus incident polarization states at 633 nm. (**a**) SEM image of one part of the fabricated metasurface hologram. Scale bar, 1 µm. Similar to Fig. 4, the figures on the right in the first (**b**–**d**) and second rows (**e**–**g**) represent target images and simulated results, respectively, and the figures in the third row (**h**–**j**) represent the corresponding experimental results.

In order to show the robustness of our proposed method for the realization of optical illusions, we also developed another metasurface device to generate Moiré fringes based on the same approach. In this case, the original target objects are two position and polarization-dependent concentric annulus. The simulated and measured results for the developed metasurface device under the illumination of incident with different polarized states are given in Fig. 6b–g, respectively. For the LCP light illumination, the left concentric annulus is located on the left side of the imaging plane (Fig. 6e,h), while the right concentric annulus are shifted on the right side under the illumination of RCP light (see Fig. 6g,j). For the LP light illumination, both of the concentric annulus are partially overlapped with each other. Moiré fringe is generated by the superposition of the light intensity of these overlapped concentric annulus, leading to the significant fishnet distribution, as shown in Fig. 6c,f,i. The calculated and measured results show good agreement, except for a slight mismatch due to the fabrication error. Unlike the optical illusion generated by the two separated “Rubin faces”, the Moiré fringe is obtained by the overlapping of two concentric annulus. In this case, the corresponding phase profile of the Moiré fringe is actually encoded onto the metasurfaces, then, the holographic image (Moiré fringe) can be reconstructed under the illumination of the LP light. Benefiting from the advantages of highly-efficient broadband reflective-type configuration and geometric metasurfaces, our designed device shows good capability to operate in the broadband. The experimental results at different wavelengths are shown in Supplementary Figure S5.

In conclusion, we have experimentally demonstrated optical illusions based on reflective metasurfaces. “Rubin faces” are realized by the geometric phase profile induced by the metasurface consisting of metallic nanorods on the top and metallic film at the bottom with the dielectric layer sandwiched between them. Upon the illumination of linearly polarized light, “Rubin’s vase” is perceived without mapping the corresponding phase profile onto the metasurface. The demonstrated metasurface devices have shown high performance in optical illusion generation with high efficiency and broad bandwidth. Our result not only provides an intuitive demonstration of the figure-ground distinction that our brains make during the visual perception, but also opens an avenue for new applications related to encryption, optical patterning, and information processing.

## Methods

### The design of holographic image

To realize a target image with a pixel array of *m* × *n* and a projection angle of *α* and *β* in the horizontal and vertical directions of the imaging plane, the period of the hologram *dx* and *dy* can be calculated by $dx=\frac{m\lambda }{2\,\tan (\alpha /2)}$ and $dy=\frac{m\lambda }{2\,\tan (\beta /2)}$, respectively. The number of pixels of the hologram is determined by *M* = *dx*/*s* and *N* = *dy*/*s*, where *s* is the pixel size of the hologram in both horizontal and vertical directions.

## Electronic supplementary material

Supplementary Information
