# Supplementary material for: Geometric Phase Generated Optical Illusion
Source: Sci Rep. 2017 Sep 12;7:11440. doi: 10.1038/s41598-017-11945-z (PMC5595789; doi:10.1038/s41598-017-11945-z)
Supplement: Supplementary file 1 — Supplementary Information [file 41598_2017_11945_MOESM1_ESM.pdf]

Supporting information for

Geometric Phase Generated Optical Illusion

Fuyong Yue[†], Xiaofei Zang[†,‡], Dandan Wen[†], Zile Li[#], Chunmei Zhang[†], Huigang Liu[†,‖], Brian D. Gerardot[†], Wei Wang[†], Guoxing Zheng[*,#], Xianzhong Chen[*,†]

[†]SUPA, Institute of Photonics and Quantum Sciences, School of Engineering and Physical Science, Heriot-Watt University, Edinburgh, EH14 4AS, UK

[‡]Shanghai Key Lab of Modern Optical System, University of Shanghai for Science and Technology, Shanghai, 200093, China

[#]School of Electronic Information, Wuhan University, Wuhan 430072, China

[‖]Tianjin Key Laboratory of Optoelectronic Sensor and Sensing Network Technology, College of Electronic Information and Optical Engineering, Nankai University, Tianjin 300350, China

Section 1. 2×2 Dammann grating design to increase the quality of holographic images.

In our experiment, the concept of Dammann grating is adopted in design to increase the fidelity of the hologram images. The difference between 2x2 array and a single period is shown in figure S1. In comparison with a single period, which produces a continuous image with lower image fidelity (i.e. more laser speckles), the 2x2 periodic hologram generates an image consisting of discrete spots. The design can be further optimized by a N x N (N is an integer) Dammann grating, which can increase the image quality sharply. However, this will in turn require the need for longer fabrication time.

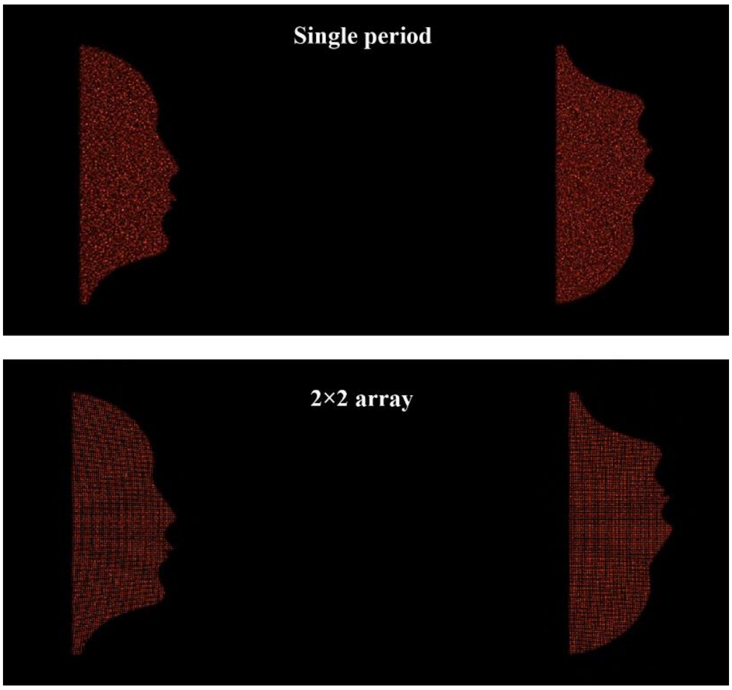

Figure S1. The reconstructed image based on phase distribution with single period and 2x2 array, respectively.

Section 2. Detailed fabrication process of nano-pattern using E-beam lithography and lift-off process.

Firstly, the positive poly methyl methacrylate resist film (PMMA 950 A2) is spin coated on the Si substrate with pre-coated gold background layer (150 nm) and the SiO_2 spacer (85 nm). The sample is baked at 180℃ on hotplate for five minutes. Then, the nanostructures are defined on the PMMA film by E-beam lithography (Raith PIONEER) under 30 kV. The exposed sample is subsequently immersed in the developer (MIBK: IPA= 1:3) for 45 s and the stopper (IPA) for 45 s. After that, the sample is raised with DI water and dried by compressed N_2 flow. Prior to gold deposition, a titanium layer of ~3 nm is deposited on the silicon dioxide (SiO_2) layer for adhesion purpose. A 30 nm gold film is deposited on the sample via electron beam evaporation. Finally, the metasurface structure is achieved by a subsequent lift-off procedure.

Section 3. The images of the inverted illusions

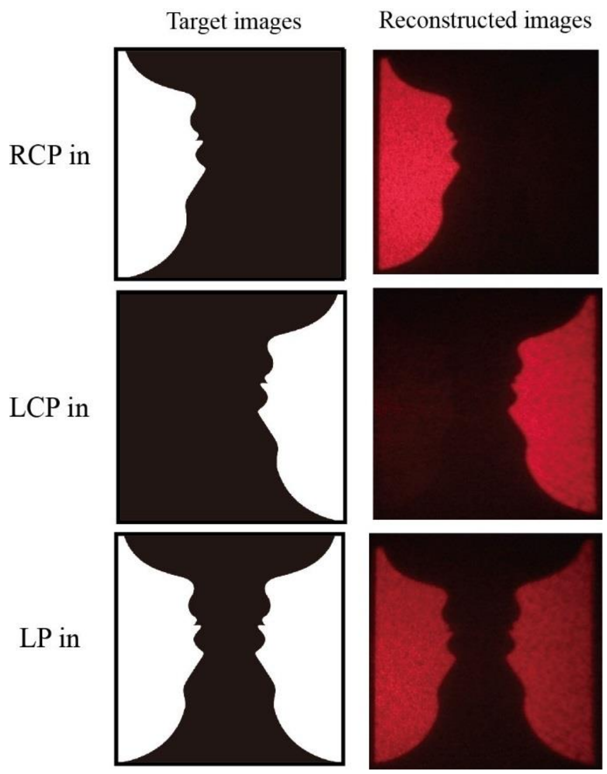

Figure S2. The target and the reconstructed inverted images versus incident polarization states at 633 nm.

Section 4. The dependence of conversion efficiency and SNR on the wavelength and polarization state of the incident light

To further characterize the metasurface, we measured the efficiency with different polarizations of the incident light at wavelength of 800 nm. The results are shown in Table S1. From the results we can see that the conversion efficiency remains by varying the polarization state of incident light.

Table S1. The measured efficiency of metasurface at different polarization states

Polarization	Left circular	Left elliptical	Linear	Right elliptical	Right circular
Efficiency	50%	49.8%	49.8%	50.9%	50.6%

We also measured the SNR at different wavelengths and polarizations which is given in in Figure S3. The fidelity and the SNR of the reconstructed images get worse at shorter wavelengths. Moreover, the SNR is higher for the circular polarization compared with that for the linear polarization.

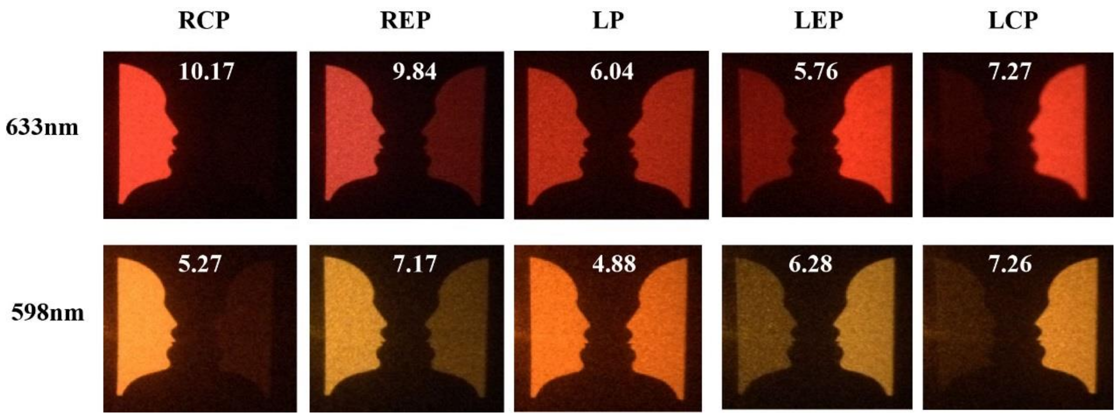

Figure S3. The measured SNR at different wavelengths and polarizations.

Section 5. The simulated efficiency of single pixel of reflective metasurface

The efficiency of single pixel of metasurface was simulated using CST microwave studio software. The three-layer structure was modelled in CST (see the inset in Fig. S4). The length, width and thickness of the rod are 220 nm, 80 nm, and 30 nm,

respectively. The thickness of gold ground layer and SiO_2 spacer layer are 150 nm and 85 nm, respectively. The refractive index of SiO_2 is 1.45. The Drude model was used for material parameters of gold, where Epsilon infinite is 1, the plasma frequency is 1.37×10^{16} *rad/s*, and the collision efficiency is 1.215×10^{14} *rad/s*. The periodic boundary condition was applied in both the *x* and *y* directions. In the simulation, the *x* and *y* polarized plane wave were normally incident onto a single nanorod, respectively. The spectra of reflection coefficients r_{xx} (*x* polarization in, *x* polarization out), r_{xy} (*x* polarization in, *y* polarization out), r_{yy} (*y* polarization in, *y* polarization out), r_{yx} (*y* polarization in, *x* polarization out) were obtained from the simulation(*1*). From the reflection coefficients of linear polarized light, the reflection coefficients for circularly polarized light can be deduced as $r_{RR} = ((\mathrm{r_{xx}}+\mathrm{r_{yy}})+\mathrm{i}\times(\mathrm{r_{xy}}-\mathrm{r_{yx}}))/2$, $r_{RL} = ((\mathrm{r_{xx}}-\mathrm{r_{yy}})+\mathrm{i}\times(\mathrm{r_{xy}}+\mathrm{r_{yx}}))/2$. r_{RR} refers to the reflection coefficient of the RCP light under the illumination of RCP light. r_{RL} refers to the reflection coefficient of the LCP light under the illumination of RCP light. The conversion efficiency is defined as the square of r_{RL}. Figure S2 shows the simulated results of the conversion efficiency by plotting r_{RR}^2 and r_{RL}^2. It should be noted that the Titanium adhesion layer is not added in this simulation.

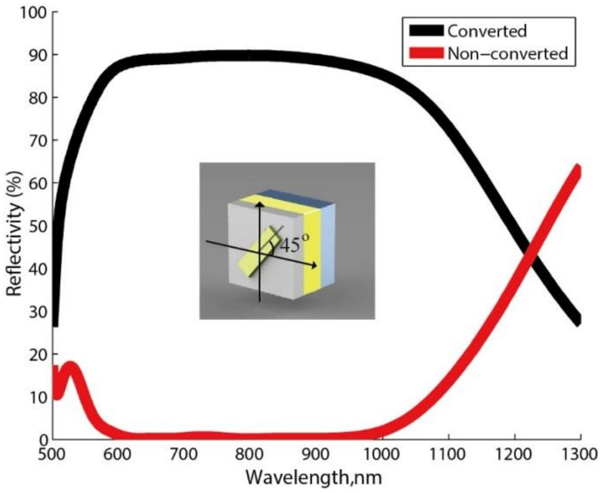

Figure S4. The simulated conversion efficiency of single pixel of reflective metasurface.

Section 6. Experimentally observed reconstructed images at different wavelengths.

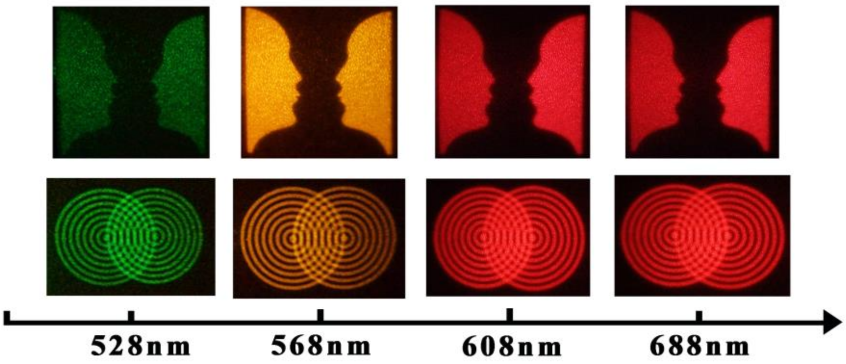

Figure S5. Experimentally reconstructed images of optical illusion and Moiré fringe at other wavelengths. The wavelengths of the incident beams are 528 nm, 568 nm, 608 nm, and 688 nm, respectively.

Reference

1. G. X. Zheng *et al.*, Metasurface holograms reaching 80% efficiency. *Nat. Nanotechnol.* **10**, 308-312 (2015).
